# Adaptation across the 2D population code explains the spatially distributive nature of motor learning

**DOI:** 10.1371/journal.pcbi.1013041

**Published:** 2025-06-04

**Authors:** Jana Masselink, Markus Lappe

**Affiliations:** Institute for Psychology and Otto Creutzfeldt Center for Cognitive and Behavioral Neuroscience, University of Münster, Münster, Germany; Johns Hopkins University, UNITED STATES OF AMERICA

## Abstract

In current computational models on oculomotor learning ‘the’ movement vector is adapted in response to targeting errors. However, for saccadic eye movements, learning exhibits a spatially distributive nature, i.e. it transfers to surrounding positions. This adaptation field resembles the topographic maps of visual and motor activity in the brain and suggests that learning does not act on the population vector but already on the level of the 2D population response. Here we present a population-based gain field model for saccade adaptation in which sensorimotor transformations are implemented as error-sensitive gain field maps that modulate the population response of visual and motor signals and of the internal saccade estimate based on corollary discharge (CD). We fit the model to saccades and visual target localizations across adaptation, showing that adaptation and its spatial transfer can be explained by locally distributive learning that operates on visual, motor and CD gain field maps. We show that 1) the scaled locality of the adaptation field is explained by population coding, 2) its radial shape is explained by error encoding in polar-angle coordinates, and 3) its asymmetry is explained by an asymmetric shape of learning rates along the amplitude dimension. Learning exhibits the highest peak rate, the widest spatial extension and a pronounced asymmetry in the motor domain, while in the visual and the internal saccade domain learning appears more localized. Moreover, our results suggest that the CD-based internal saccade representation has a response field that monitors only part of the ongoing saccade changes during learning. Our framework opens the door to study spatial generalization and interference of learning in multiple contexts.

## Author summary

Understanding the neural mechanisms that enable adaptive motor behavior remains a significant challenge in sensorimotor control. First, we show how learning associated with a specific target position spreads across the visual field. Second, we show how learning acts on the two-dimensional population code for visual, motor and internal movement representations. Third, our model dissociates the different sites of the oculomotor circuitry that plastically adapt to motor errors and serve the optimization of future movements. In sum, our framework sets the stage to study how the spatially distributive representation of visuomotor signals shapes various adaptation phenomena, like position-dependent adaptation in multiple target directions or sequential learning at differentes sites in the visual field.

## Introduction

Human motor behavior is highly adaptive and strives for optimization [1, 2]. A popular paradigm to study the plasticity of the motor system is adaptation to an external perturbation that is assigned to an internal sensorimotor failure [3–5]. During adaptation of saccadic eye movements, a saccade target is shifted during movement execution such that the saccade vector adapts trial by trial to minimize the resulting motor error [6–8]. Current computational models on saccade adaptation capture various aspects of this learning process, like error sensitivity [9–11], motor memory [12], characteristics of the target step [13] and accompanying changes in visuospatial target localization [14, 15]. All these models use a spatially one-dimensional framework in which adaptation acts on a single movement vector for a specific goal position in the visual field. However, adaptation of saccadic eye movements has a spatially distributive nature, i.e. it transfers to target positions in the surrounding [7, 16, 17]. Frens & Van Opstal (1994) [18] have called this the adaptation field as it resembles how motor-related activity is represented in the brain, e.g. by the movement fields in the SC [19–21].

The structure of the adaptation field exhibits four essential characteristics. 1) The adaptation field is scaled, i.e. the transfer is a descending function of the distance to the adaptation position [16, 18, 22]. 2) The adaptation field is radial. This means that the change of the saccade vector – and not the change in the horizontal and vertical component – is transferred to other saccades [7, 17, 23]. For example, if a horizontal saccade adapts to a horizontal inward target step, the adaptation transfer to an oblique saccade shortens the entire saccade vector while keeping its direction stable. By contrast, if the transfer were based on the horizontal and vertical components separately, only the horizontal component of the oblique saccade would shorten, altering its direction, but this is not the case. 3) The adaptation field is asymmetric along the amplitude dimension. Hence, the transfer is higher for targets that are outward than for targets that are inward of the adaptation position [17, 24–26]. 4) Changes in pre- and trans-saccadic visual target localization that accompany saccade adaptation show an adaptation field as well [22, 25]. The change in the pre-saccadic visual target localization shows that not only the motor command is updated throughout adaptation but also the visuospatial representation of the saccade goal itself [14, 27, 28]. The change of the trans-saccadic visual target localization suggests that also the internal representation of the saccade based on corollary discharge (CD) [29] is updated throughout adaptation [14, 15].

The existence of the adaptation field means that learning occurs simultaneously for target locations close to the adaptation position, i.e. for neurons that are tuned to saccades of similar amplitude and direction. This suggests that learning already acts on the level of the response distribution across a neural population that collectively encodes the amplitude and direction of a saccade. Population coding mechanisms are known form the retinotopic movement fields in the SC [19, 20, 30] but also in other brain regions involved in oculomotor control, like the frontal eye fields (FEF) [31–33], parietal cortex [34, 35] and the cerebellum [36].

Here we present a population-coded model for the adaptation of saccade amplitudes in which learning acts on three gain field maps for visual, motor and the internal saccade representation. In each of these maps, a gain field centered at the saccade target modulates the responses at neighboring portions in the map, thus changing the population response over the course of adaptation. Note that our use of gain fields for adaptation is different from the use of gain fields for coordinate transformations and spatial updating, areas where gain field models have also been applied with great success [37–41]. We fit the model to saccade amplitudes and visual target localizations during adaptation and demonstrate 1) through which mechanisms the model explains the scaled, polar and asymmetric shape of the adaptation field, 2) how visual, motor and CD learning rates are spatially distributed across the visual field, and 3) that the CD-based internal saccade estimate has a response field that underestimates the ongoing saccade changes during learning. Our findings support the idea that learning operates on the level of a neural population that collectively encodes saccade amplitude and direction.

## Results

The model describes amplitude adaptation of conjugate saccades in any direction within the 360 degree range across the fronto-parallel plane. Each signal is represented by a population response across the two-dimensional retino-centric field. Locations are encoded by the identity of the active population, i.e. by computing a population vector across the population response. Based on Masselink & Lappe (2021) [14], adaptation operates at three sites of the oculomotor circuitry, i.e. the sensorimotor transformations between visual, motor and internal movement representations. In the model at hand, these sensorimotor transformations are implemented as visual, motor and CD gain field maps that scale the population response of the respective signal and learn from error that is encoded in polar-angle coordinates, i.e. as a directed amplitude error.

### Model framework

**Fig 1:**
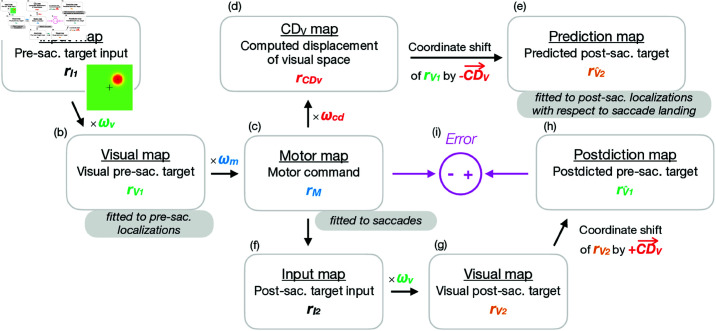
Model framework. (a) The population response $r_{I_{1}}$ to the pre-saccadic target on the input map is defined by a two-dimensional Gaussian distribution. (b) The activity on the input map is routed into the visual map where the visual population response $r_{V_{1}}$ is scaled by the visual gain field $\omega_{v}$. The population vector ${\vec{V}}_{1}$ is computed across $r_{V_{1}}$ and specifies where the target is visually localized in retinal coordinates. (c) The visual map is transformed into a motor map by the motor gain field $\omega_{m}$, resulting in the motor population response *r*_*M*_ and the motor command $\vec{M}$, i.e. in motor coordinates. (d) Before saccade start, the CD gain field $\omega_{cd}$ transforms the motor map into a ${CD}_{V}$ map with the population response $r_{CD_{V}}$. The resulting population vector ${\vec{CD}}_{V}$ defines the internal estimate of saccade size in visual coordinates. (e) A shift of the visual population response $r_{V_{1}}$ by ${\vec{CD}}_{V}$ defines the population response $r_{{\hat{V}}_{2}}$ on the prediction map that specifies ${\vec{\hat{V}}}_{2}$, i.e. where the post-saccadic target is predicted to appear on the retina after saccade landing. (f) After saccade execution, the population response $r_{I_{2}}$ to the post-saccadic target on the input map is routed into (g) the visual map by the visual gain field $\omega_{v}$. The visual population response $r_{V_{2}}$ defines the visual post-saccadic target position ${\vec{V}}_{2}$ in retinal coordinates. (h) In order to compute the error of the motor command, the oculomotor system first postdicts the visual population response to the post-saccadic target back to pre-saccadic space. Hence, $r_{V_{2}}$ is shifted by ${\vec{CD}}_{V}$, resulting in the population response $r_{{\hat{V}}_{1}}$ on the postdiction map. (i) The postdictive motor error $\vec{E}$ is computed as the error of the motor command $\vec{M}$ with respect to the postdicted target position ${\vec{\hat{V}}}_{1}$.

Fig 1 presents the model framework. On the input map, the population response $r_{I_{1}}$ to the pre-saccadic target is described by a two-dimensional Gaussian distribution (see Fig 1a). Hence, the input map provides a place code representation of the pre-saccadic target position in retinal coordinates. The activity on the input map is routed into the visual map where the visual population response $r_{V_{1}}$ is scaled by the visual gain field $\omega_{v}$ (Fig 1b). The motor gain field $\omega_{m}$ transforms the visual map into a motor map, leading to the motor population response *r*_*M*_ (Fig 1c). Before saccade onset, the CD gain field $\omega_{cd}$ transforms the motor representation into a visual representation of the expected saccade size, i.e. the ${\text{CD}}_{\text{V}}$ map with the population response $r_{CD_{V}}$ (Fig 1d).

The visual, motor and the CD gain fields are implemented as a one-to-one connectivity structure between corresponding positions in two maps, where each position in the respective input map connects exclusively to the corresponding position in the respective output map. The one-to-one connectivity structure ensures that the gain fields, i.e. reference frame transformations, and its change across adaptation are explicit and analytically accessible. Based on the one-to-one connective structure, the gain fields operate as a rate code on the population responses, i.e. they scale the activity independently at each position on the map, and this differential scaling across the map affects the overall population response. In contrast to a place code mechanism, where the center of activity is shifted spatially on the map, the gain fields modulate the magnitude of activity at fixed positions, indirectly shaping the population response and the resulting population vector. The population vector is read out from these maps as the sum of the active population, leading to the visual pre-saccadic target representation ${\vec{V}}_{1}$, the motor command $\vec{M}$ and the computed displacement of visual space ${\vec{CD}}_{V}$, i.e. the internal estimate of how large the saccade will be.

The motor gain field $\omega_{m}$ acts as an inverse model that transforms a visual representation into a motor representation. If the whole motor gain field is equal to 1, i.e. if it is perfectly tuned, the saccade will land on the visual pre-saccadic target location. The CD gain field $\omega_{cd}$ acts as a forward dynamics model that re-transforms the motor representation into a visual representation. If the whole CD gain field $\omega_{cd}$ is equal to 1, it is perfectly tuned such that ${\vec{CD}}_{V}$ matches the actual saccade size.

Before saccade execution, the visuomotor system predicts where the target will appear on the retina after saccade landing, i.e. by spatial updating. Hence, the visual population response $r_{V_{1}}$ is shifted by the internal saccade estimate ${\vec{CD}}_{V}$. The resulting population response $r_{{\hat{V}}_{2}}$ on the prediction map specifies the predicted post-saccadic target in retinal coordinates ${\vec{\hat{V}}}_{2}$ (Fig 1e). After saccade landing, the visual gain field transforms the population response $r_{I_{2}}$ to the actual post-saccadic target on the input map (Fig 1f) into the visual population response $r_{V_{2}}$ (Fig 1g). To compute the error of the motor command $\vec{M}$, the oculomotor system uses the visual post-saccadic target representation to postdict where the target was in pre-saccadic coordinates. Hence, $r_{V_{2}}$ is postdicted back to pre-saccadic space based on a backward coordinate shift by ${\vec{CD}}_{V}$, resulting in the population response $r_{{\hat{V}}_{1}}$ on the postdiction map (Fig 1h). The postdictive motor error $\vec{E}$ is then computed as the error of the motor command $\vec{M}$ with respect to the postdicted target position ${\vec{\hat{V}}}_{1}$, i.e. $\vec{E}$ = ${\vec{\hat{V}}}_{1}-\vec{M}$ (Fig 1i).

### Model simulations for inward and outward adaptation of saccades in different directions

**Fig 2:**
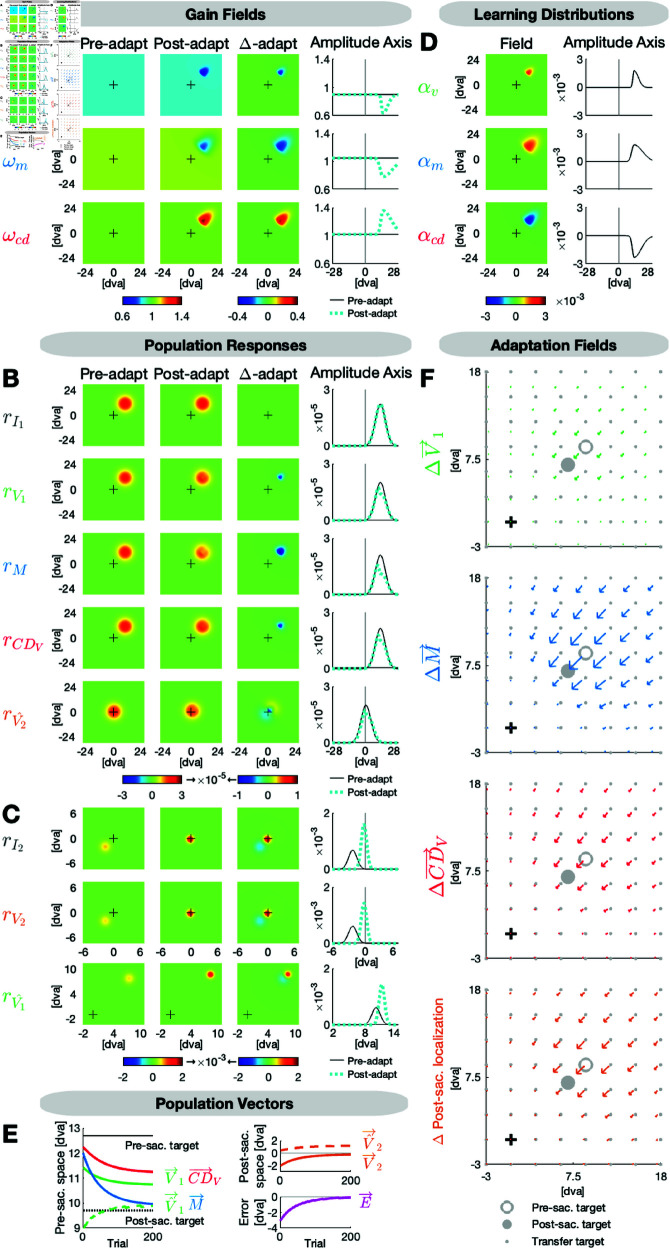
Model simulation for inward adaptation of an oblique saccade to the right hemifield. The target is presented 12.7 dva to the upper right and steps 3 dva inward during saccade execution. **(A)** The visual, motor and CD gain fields $\omega_{v}$, $\omega_{m}$ and $\omega_{cd}$ are well calibrated before adaptation ($\omega_{v_{C}}$ = 0.900, $\omega_{m_{C}}$ = 1.050, $\omega_{{cd}_{C}}$ = 1.020). Inward adaptation causes a local indentation in $\omega_{v}$ and $\omega_{m}$ and a local elevation in $\omega_{cd}$ around the adaptation target position. **(B)** Due to the accurate calibration in the pre-adaptation state, the input map $r_{I_{1}}$ and the visual, motor and ${CD}_{V}$ maps $r_{V_{1}}$, *r*_*M*_ and $r_{CD_{V}}$ look very similar. Hence, the prediction map $r_{{\hat{V}}_{2}}$ expects the post-saccadic target to appear in the fovea. **(C)** Due to the peri-saccadic inward target step, on the input map $r_{I_{2}}$ and the visual map $r_{V_{2}}$, the post-saccadic target appears in the lower left quadrant with respect to the fovea. The actual location of the post-saccadic target is used to update the pre-saccadic target position on the postdiction map $r_{{\hat{V}}_{1}}$. **(D)** The learning distributions $\alpha_{v}$, $\alpha_{m}$ and $\alpha_{cd}$ determine the rate of learning for each position in the visual field. They exhibit an asymmetric shape along the amplitude dimension with a prolonged expansion into the periphery ($\phi_{v}$ = 0.002, $\phi_{m}$ = 0.002, $\phi_{cd}$ = -0.002, $\sigma_{v_{F}}$ = 1.00, $\sigma_{m_{F}}$ = 2.00, $\sigma_{{cd}_{F}}$ = 1.50, $\sigma_{v_{P}}$ = 3.50, $\sigma_{m_{P}}$ = 7.00, $\sigma_{{cd}_{P}}$ = 6.00, $\sigma_{v_{O}}$ = 2.00, $\sigma_{m_{O}}$ = 4.00, $\sigma_{{cd}_{O}}$ = 3.00). **(E)** The visual pre-saccadic target ${\vec{V}}_{1}$, the motor command $\vec{M}$ and the internal saccade estimate ${\vec{CD}}_{V}$ adapt to minimize the postdictive motor error $\vec{E}$. Further depicted signals are the postdicted pre-saccadic target ${\vec{\hat{V}}}_{1}$, the predicted post-saccadic target ${\vec{\hat{V}}}_{2}$ and the visual post-saccadic target ${\vec{V}}_{2}$. **(F)** The visual, motor and ${CD}_{V}$ adaptation fields and the post-saccadic localization exhibit a scaled, radial and asymmetric shape.

Fig 2 presents a model simulation about 200 adaptation trials with a 12.7 dva pre-saccadic target to the upper right with 45 angular degree. During saccade execution, the target is stepped 3 dva inward, i.e. against saccade direction.

Fig 2A depicts the state of the gain fields $\omega_{v}$, $\omega_{m}$ and $\omega_{cd}$ on the first trial (Pre-adapt, column 1), on the last trial (Post-adapt, column 2), the change from pre- to post-adaptation (Δ-adapt, column 3) as well as the pre- and post-adaptation states along the amplitude axis (column 4). Before adaptation, each gain field is described as a flat plane, with activity levels constant across the entire map. As the visuomotor gain fields are well calibrated before adaptation (i.e. close to 1), the population responses $r_{I_{1}}$, $r_{V_{1}}$, *r*_*M*_ and $r_{{CD}_{V}}$ look very similar in the pre-adaptation state (Fig 2B). Small gain field deviations from 1 become more apparent in the population vectors (Fig 2E), showing, in this example, a slight underestimation of the pre-saccadic target eccentricity ${\vec{V}}_{1}$ (in line with [14, 22, 25]), a hypometric saccade $\vec{M}$ (in line with [42–44]) and a fairly accurate internal saccade estimate ${\vec{CD}}_{V}$ at the start of adaptation.

Due to the accurate calibration of visuomotor gains in the pre-adaptation state, the oculomotor system predicts the post-saccadic target to appear in the fovea after saccade landing ($r_{{\hat{V}}_{2}}$ in Fig 2B, ${\vec{\hat{V}}}_{2}$ in Fig 2E). However, due to the peri-saccadic inward target step, the target appears in the lower left quadrant with respect to the fovea ($r_{I_{2}}$ and $r_{V_{2}}$ in Fig 2C, ${\vec{V}}_{2}$ in Fig 2E). The population responses of the post-saccadic signals are higher but narrower than those of the pre-saccadic signals as the post-saccadic target is less eccentric than the pre-saccadic target. Due to the lower left position of the post-saccadic target, the postdicted target ${\vec{V}}_{1}$ is localized closer to the fovea in pre-saccadic coordinates (Fig 2C, 2E). Consequently, the postdictive motor error $\vec{E}$ is directed inward, i.e. against saccade direction (Fig 2E).

The visuomotor gain fields $\omega_{v}$, $\omega_{m}$ and $\omega_{cd}$ learn to nullify the postdictive motor error $\vec{E}$ along the amplitude dimension, resulting in a trial-by-trial shortening of the saccade vector $\vec{M}$ as well as a trial-by-trial inward shift of the visual pre-saccadic target position ${\vec{V}}_{1}$ and ${\vec{CD}}_{V}$ until $\vec{E}$ is nullified (Fig 2E). Learning occurs locally around the pre-saccadic target position. Thereby, the learning distributions $\alpha_{v}$, $\alpha_{m}$ and $\alpha_{cd}$ (Fig 2D) determine the learning rate for each position across the gain fields $\omega_{v}$, $\omega_{m}$ and $\omega_{cd}$. Hence, they specify how much each position within each gain field learns from error on a given trial. The local elevation within $\alpha_{v}$ and $\alpha_{m}$ means that a postdictive motor error $\vec{E}$ < 0 along the amplitude dimension, i.e. encoding an inward error, will lead to a local decrease of $\omega_{v}$ and $\omega_{m}$. The local indentation within $\alpha_{cd}$ means that a postdictive motor error $\vec{E}$ < 0 along the amplitude dimension will lead to a local increase of $\omega_{cd}$. The $\alpha_{cd}$ learning direction is reversed because $\omega_{cd}$, acting as a forward dynamics model, is the antagonist of $\omega_{m}$, acting as an inverse model. As a consequence, the downscaled population response in *r*_*M*_ is upscaled in $r_{CD_{V}}$ (Fig 2B), which, overall, leads to an underestimation of the actual saccade change $\vec{M}$ by ${\vec{CD}}_{V}$ (Fig 2E, congruent with [14] and [15]).

The learning distributions $\alpha_{v}$, $\alpha_{m}$ and $\alpha_{cd}$ are symmetrically shaped along the orthogonal amplitude axis and asymmetrically shaped along the amplitude axis (Fig 2D). Thereby, the learning rate falls sharply for positions inward of the adaptation target, i.e. closer to the fovea, and more gradual for positions outward of the adaptation target. This results in non-uniform adaptation fields of the visual pre-saccadic target position ${\vec{V}}_{1}$, the motor command $\vec{M}$ and the computed displacement of visual space ${\vec{CD}}_{V}$. Fig 2F presents these adaptation fields, i.e. the change of the signals from the pre- to the post-adaptation state. Consistent with Collins *et al*. (2007) [25], Schnier *et al*. (2010) [22], Masselink & Lappe (2021) [14] and Masselink *et al*. (2023) [15], there is most adaptation and most transfer to other positions in the motor command $\vec{M}$, i.e. the saccade vector, a medium change in ${\vec{CD}}_{V}$, and a rather small change in the visual pre-saccadic target position ${\vec{V}}_{1}$. The amount of learning, its spatial distribution as well as its asymmetry along the amplitude dimension are determined by the shape of the learning distributions $\alpha_{v}$, $\alpha_{m}$ and $\alpha_{cd}$. In addition, in the lower right plot (ΔPost-saccadic localization), we present the expected change in an experimental task in which subjects have to visually localize after saccade landing where the pre-saccadic target was (see Results and Methods below).

When the motor command $\vec{M}$ has converged to a new steady state, the saccade lands close to (but not on) the post-saccadic target (Fig 2E; as known from [7, 17, 45, 46]) and the postdictive motor error $\vec{E}$ is nullified.

**Fig 3:**
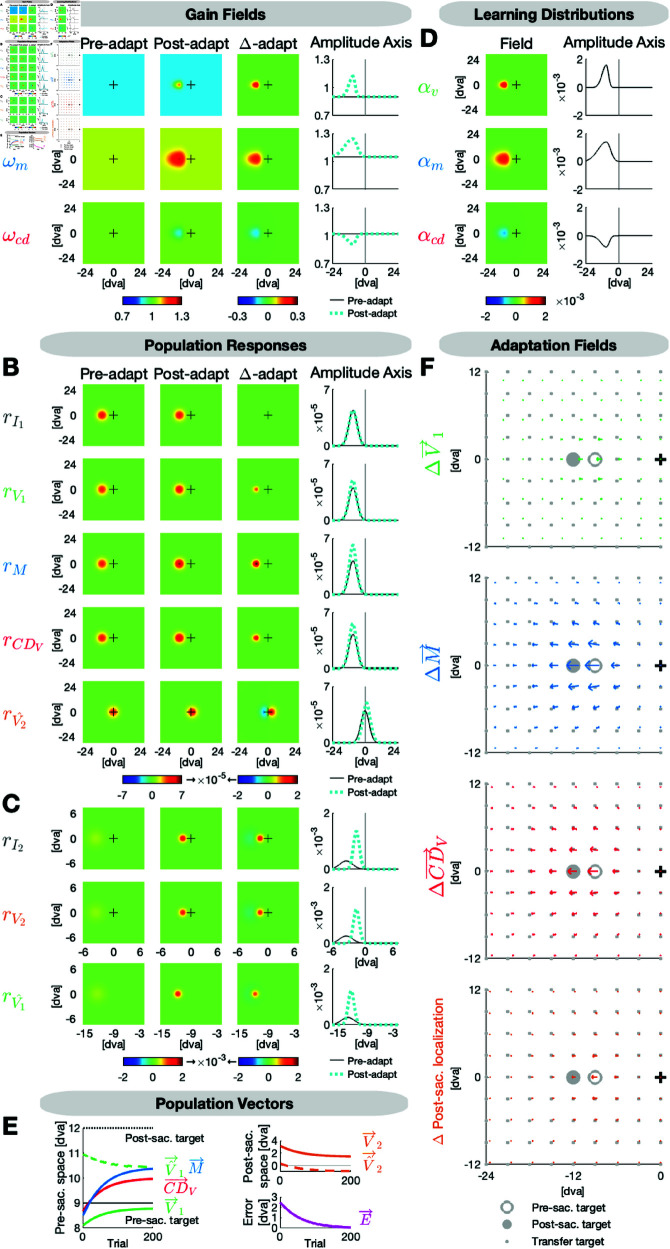
Model simulation for outward adaptation of a horizontal saccade to the left hemifield. The target is presented 9 dva to the left and steps 3 dva outward during saccade execution. **(A)** The visual, motor and CD gain fields $\omega_{v}$, $\omega_{m}$ and $\omega_{cd}$ are well calibrated before adaptation ($\omega_{v_{C}}$ = 0.900, $\omega_{m_{C}}$ = 1.050, $\omega_{{cd}_{C}}$ = 1.020). Outward adaptation causes a local elevation in $\omega_{v}$ and $\omega_{m}$ and a local indentation in $\omega_{cd}$ around the adaptation target position. **(B)** Due to the accurate calibration in the pre-adaptation state, the input map $r_{I_{1}}$ and the visual, motor and ${CD}_{V}$ maps $r_{V_{1}}$, *r*_*M*_ and $r_{CD_{V}}$ look very similar. Hence, the prediction map $r_{{\hat{V}}_{2}}$ expects the post-saccadic target to appear in the fovea. **(C)** Due to the peri-saccadic outward target step, on the input map $r_{I_{2}}$ and the visual map $r_{V_{2}}$, the post-saccadic target appears on the left horizontal meridian with respect to the fovea. The actual location of the post-saccadic target is used to update the pre-saccadic target position on the postdiction map $r_{{\hat{V}}_{1}}$. **(D)** The learning distributions $\alpha_{v}$, $\alpha_{m}$ and $\alpha_{cd}$ determine the rate of learning for each position in the visual field. They exhibit an asymmetric shape along the amplitude dimension with a prolonged expansion into the periphery ($\phi_{v}$ = 0.002, $\phi_{m}$ = 0.001, $\phi_{cd}$ = -0.001, $\sigma_{v_{F}}$ = 1.50, $\sigma_{m_{F}}$ = 3.00, $\sigma_{{cd}_{F}}$ = 2.00, $\sigma_{v_{P}}$ = 3.00, $\sigma_{m_{P}}$ = 6.00, $\sigma_{{cd}_{P}}$ = 4.00, $\sigma_{v_{O}}$ = 2.00, $\sigma_{m_{O}}$ = 4.00, $\sigma_{{cd}_{O}}$ = 3.00). **(E)** The visual pre-saccadic target ${\vec{V}}_{1}$, the motor command $\vec{M}$ and the internal saccade estimate ${\vec{CD}}_{V}$ adapt to minimize the postdictive motor error $\vec{E}$. Further depicted signals are the postdicted pre-saccadic target ${\vec{\hat{V}}}_{1}$, the predicted post-saccadic target ${\vec{\hat{V}}}_{2}$ and the visual post-saccadic target ${\vec{V}}_{2}$. **(F)** The visual, motor and ${CD}_{V}$ adaptation fields and the post-saccadic localization exhibit a scaled, radial and asymmetric shape.

While Fig 2 demonstrated inward adaptation of an oblique saccade to the right hemifield, we show an example simulation for outward adaptation of a horizontal saccade to the left hemifield in Fig 3. We choose different adaptation directions, saccade directions, and hemifields to demonstrate the model’s functionality across a broad range of possible situations, i.e. amplitude adaptation in both directions across the full 360 degree saccade range. In the simulation shown in Fig 3, the pre-saccadic target is placed 9 dva to the left on the horizontal meridian, and the target is stepped 3 dva outward during saccade execution, i.e. in saccade direction. In this case, the postdictive motor error $\vec{E}$ is > 0 along the amplitude dimension (Fig 3E), teaching the visual gain field $\omega_{v}$ and the motor gain field $\omega_{m}$ to locally increase and the CD gain field $\omega_{cd}$ to locally decrease specified by the learning distributions $\alpha_{v}$, $\alpha_{m}$ and $\alpha_{cd}$ (Fig 3A, 3D). This leads to a gradual increase of the motor command $\vec{M}$, i.e. to saccade lengthening, and to an outward shift of the visual pre-saccadic target position ${\vec{V}}_{1}$. Consistent with with Masselink & Lappe (2021) [14], ${\vec{CD}}_{V}$ reflects a lengthening of the saccade but still underestimates its size. Moreover, in line with Kojima *et al*. (2004) [47], Panouilleres *et al*. (2008) [48] and Pelisson *et al*. (2010) [26], outward adaptation converges with a larger remaining distance between the saccade landing location and the post-saccadic target than inward adaptation (see motor command $\vec{M}$ and ${\vec{V}}_{2}$ in Fig 3E compared to Fig 2E). Fig 3B–3C shows the population responses of the pre- and the post-adaptation state as well as the change across adaptation.

### Population coding, polar-angle error and asymmetric learning distributions explain the spatial layout of the adaptation field

The adaptation field model reflects essential properties of oculomotor learning and its transfer to spatial positions in the surrounding (Figs 2F and 3F). 1) The adaptation field is scaled with most adaptation at the primary target position and less adaptation at neighboring target positions (in line with [16, 18, 22]). The model produces this scaled locality because learning is implemented to act on the population code representation of visuomotor signals. 2) The adaptation field is radial such that learning acts on the amplitude representation of the saccade and not separately on the horizontal and vertical saccade components (consistent with [7, 17, 23]). For example, in Fig 3, outward adaptation of horizontal leftward saccades produces oblique saccade changes for targets in the upper and in the lower part of the visual field, rather than isolated horizontal changes as it would be the case if adaptation transfer were component-specific. In Fig 2, inward adaptation of saccades to the upper right produce oblique saccade changes on both sides of the amplitude axis. The oblique shape is reproduced because in the model, learning is implemented to operate in polar-angle coordinates, i.e. by a directed amplitude-error instead of a horizontal and a vertical error component (for polar-angle encoding see [49, 50]). This is in line with the notion that learning operates in oculomotor areas where saccades are represented by their amplitude and direction and not in more peripheral structures, i.e. in the brain stem, where saccade representations are split into vertical and horizontal components before being sent to the eye muscles (in line with [23, 24, 51]). 3) The adaptation field is asymmetric along the amplitude dimension, i.e. the amount of transfer falls more sharply for targets inward of the primary target position and more gradually for targets outward of the primary target position (Figs 2F and 3F; consistent with [17, 24–26]). In the model, this asymmetry is explained by an asymmetric distribution of learning rates along the amplitude axis. 4) Not only the saccade vector but also the pre- and post-saccadic target localizations and ${\vec{CD}}_{V}$ show an adaptation field that is scaled, radial and asymmetric [14, 15, 27, 28]. In Figs 2F and 3F, ${\vec{V}}_{1}$ is equivalent to where subjects would localize a briefly flashed target during fixation (pre-saccadic target localization) and $\vec{M}$ is equivalent to the saccade vector.

### Spatial transfer occurs for visual, motor and internal saccade representation

**Fig 4:**
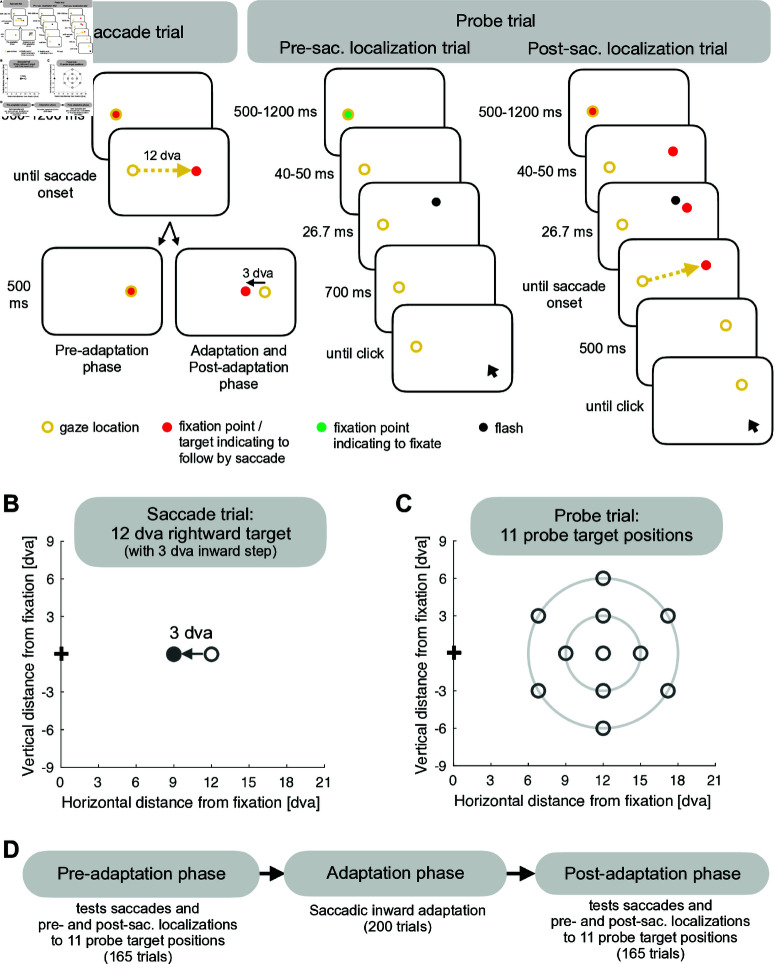
Experimental design. **(A)** A saccade trial was performed with a 12 dva rightward stepping target, that, from the adaptation phase onwards, jumped 3 dva inward during saccade execution. In a pre-saccadic localization trial, a stimulus was briefly flashed for 26.7 ms at one of 11 probe positions. Subjects had to keep their gaze fixated at the now invisible fixation position while localizing where they had perceived the flash with a mouse cursor. In a post-saccadic localization trial, the fixation target stepped to one of the 11 probe positions. After 40-50 ms, a stimulus was flashed for 26.7 ms nearby. Following saccade execution, subjects had to stay fixated at the saccade landing location while localizing where they had perceived the flash with a mouse cursor. In case their gaze deviated more than 4 dva, a beep tone reminded subjects to stay fixated at the saccade landing position (or at the fixation position in pre-saccadic localization trials). Please note that the yellow circle indicates eye position and was not visible on the screen. **(B)** In a saccade trial, the target was presented 12 dva to the right on the horizontal meridian with a 3 dva inward step during saccade execution from the adaptation phase onwards. **(C)** The probe targets were arranged on two invisible concentric circles with a radius of 3 dva and 6 dva around the adaptation target position. **(D)** The pre-adaptation and the post-adaptation phases tested the state of saccades, pre- and post-saccadic localizations for the 11 probe positions. The adaptation phase consisted of 200 saccade trials with peri-saccadic inward target step to induce adaptation.

**Fig 5:**
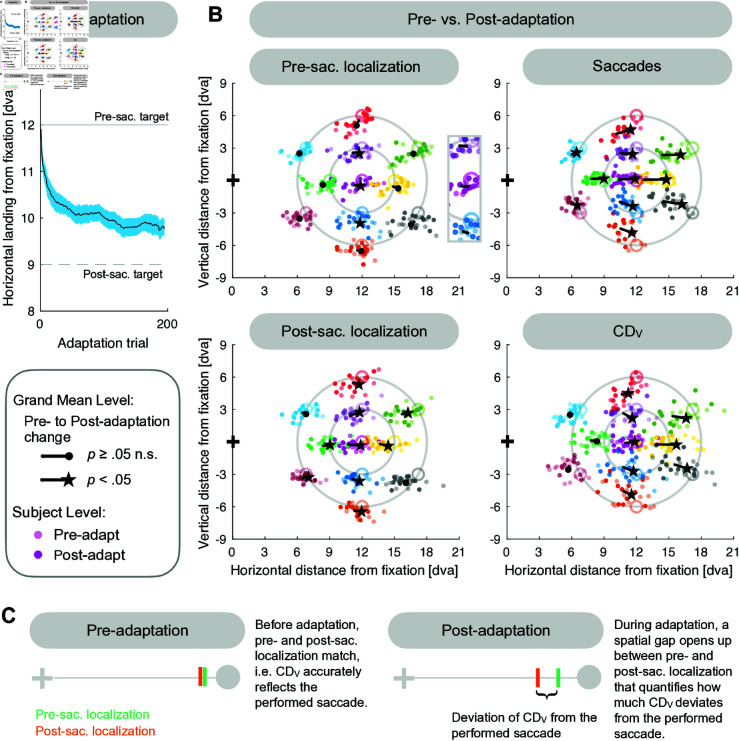
Experimental results. **(A)** Saccade amplitude shortening during the adaptation phase (subject mean ± SE). **(B)** Adaptation fields for pre-saccadic localization, post-saccadic localization, saccades and ${CD}_{V}$ (pre- to post-adaptation mean across subjects with individual subject data in the background). The saccade amplitudes show the strongest, clearly shaped adaptation field, ${CD}_{V}$ shows a medium sized adaptation field and post-saccadic localization show a smaller sized adaptation field. The adaptation effect is limited for pre-saccadic localizations with a marginally significant amplitude reduction at the adaptation target position (*p* = .053). Significant transfer can be found 3 dva above and below the adaptation target. **(C)** Illustration of ${CD}_{V}$ quantification. Before adaptation, the pre-saccadic flash is localized at the same position if judged during fixation (pre-saccadic localization) or after performing a saccade (post-saccadic localization). Hence, the spatial integration between the pre- and post-saccadic visual scene is correct such that ${CD}_{V}$ accurately reflects the saccade vector. During adaptation, a spatial gap opens up between pre- and post-saccadic localization, indicating that ${CD}_{V}$ has decoupled from the actually performed saccade.

To test the model, we experimentally measured saccade adaptation with a 12 dva rightward target that stepped 3 dva inward during saccade execution (Fig 4A–4B). Before and after adaptation, we measured saccades as well as pre- and post-saccadic target localizations for 11 probe positions, i.e. for the adaptation target and 10 positions in the surrounding (Fig 4C–4D). In pre-saccadic localization trials, a stimulus was briefly flashed at one of the 11 probe positions. Subjects had to localize with a mouse pointer where they had perceived the stimulus while still holding gaze at the fixation location (Fig 4A). This served to fit the visual pre-saccadic target position ${\vec{V}}_{1}$ at the 11 probe positions before and after adaptation. In the post-saccadic localization trials, a saccade target was presented at one of the 11 probe positions and a stimulus was briefly flashed close to the saccade target. After saccade execution to the target, the display was completely dark and subjects had to localize the perceived flash position with the mouse pointer while holding gaze at the saccade landing position (Fig 4A). If the post-saccadic target localization matches the pre-saccadic target localization, the spatial integration between the pre- and the post-saccadic visual scene is correct, i.e. the computed displacement of visual space ${\vec{CD}}_{V}$ matches the actually performed saccade. By contrast, a deviation between the pre- and the post-saccadic target localization quantifies how much ${\vec{CD}}_{V}$ deviates from the actually performed saccade (Fig 5C). Hence, the combination of pre- and post-saccadic localization trials and saccade amplitudes served to retrieve ${\vec{CD}}_{V}$ in the experimental results and to fit ${\vec{CD}}_{V}$ in the modeling results.

Fig 5A depicts the mean horizontal saccade amplitude that, starting with 11.9 ± 0.5 dva (pre-adaptation phase), was gradually shortened trial by trial by -2.4 ± 0.9 dva (pre-adaptation phase until last 8 trials of the adaptation phase, *t*(12) = -6.46, *p* < .001). Fig 5B presents the mean change from the pre- to the post-adaptation phase for saccade landing locations, pre- and post-saccadic target localizations and ${\vec{CD}}_{V}$. The saccades show a strong adaptation field, including scaling, radiality and asymmetry, and significant amplitude shortening at all probe positions (p≤ .002). The amplitude of the pre-saccadic localization exhibited a marginally significant reduction of -0.6 ± 0.9 dva at the adaptation target position (*t*(12) = -2.15, *p* = .053) and a significant reduction at the positions 3 dva above and below the adaptation target (*t*(12) = -2.93, *p* = .013 and *z* = -2.27, *p* = .023). The pre-saccadic localizations at all other positions did not show substantial adaptation (p≥ .008). Hence, the adaptation effect of the pre-saccadic localization is small and its transfer is mainly local. The adaptation of the post-saccadic localization and its transfer was more pronounced with a significant change of -1.2 ± 0.6 dva at the adaptation target (*t*(12) = -7.64, *p* < .001) and significant changes at eight other probe locations (p≤ .013; the remaining two positions were not significant with p≥ .153). The ${CD}_{V}$ signal demonstrates an adaptation field with a significant amplitude shortening of -0.9 ± 1.0 dva at the adaptation target location (*t*(12) = -3.20, *p* < .008) and for all probe positions above, below and outward of the adaptation target (p≤ .047). ${\vec{CD}}_{V}$ changes at the three probe positions inward of the adaptation target did not show significant effects (p≥ .139).

### The distribution of learning differs between gain field maps

**Fig 6:**
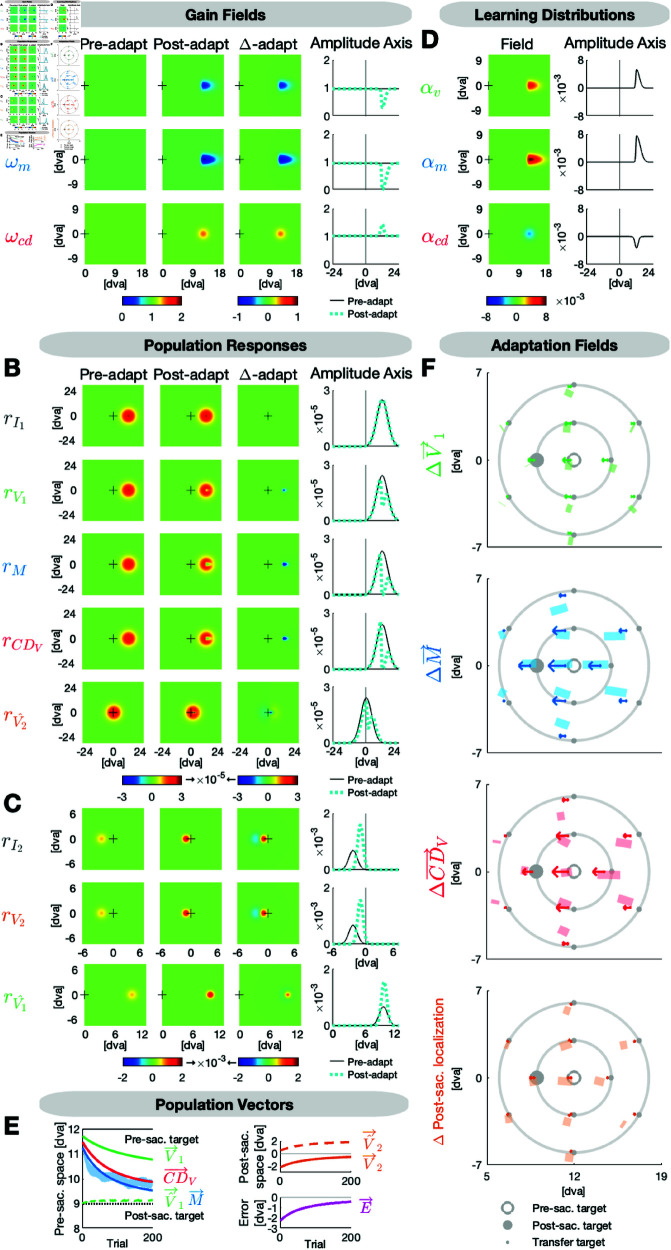
Model fit to the adaptation data. The target is presented 12 dva to the right and steps 3 dva inward during saccade execution. **(A)** The visual, motor and CD gain fields $\omega_{v}$, $\omega_{m}$ and $\omega_{cd}$ are well calibrated before adaptation. Inward adaptation causes a local indentation in $\omega_{v}$ and $\omega_{m}$ and a local elevation in $\omega_{cd}$ around the adaptation target position. **(B)** Due to the accurate calibration in the pre-adaptation state, the input map $r_{I_{1}}$ and the visual, motor and ${CD}_{V}$ maps $r_{V_{1}}$, *r*_*M*_ and $r_{CD_{V}}$ look very similar. Hence, the prediction map $r_{{\hat{V}}_{2}}$ expects the post-saccadic target to appear in the fovea. **(C)** Due to the peri-saccadic inward target step, on the input map $r_{I_{2}}$ and the visual map $r_{V_{2}}$, the post-saccadic target appears on the left horizontal meridian with respect to the fovea. The actual location of the post-saccadic target is used to update the pre-saccadic target position on the postdiction map $r_{{\hat{V}}_{1}}$. **(D)** The shape of the learning distributions $\alpha_{v}$, $\alpha_{m}$ and $\alpha_{cd}$ differs between gain field maps. The motor learning distribution $\alpha_{m}$ exhibits the highest peak rate, the widest spatial span and the largest asymmetry along the amplitude dimension, i.e. it largely expands into the periphery. The asymmetry is followed by the smaller and less peaked visual learning distribution $\alpha_{v}$. The CD learning distribution $\alpha_{cd}$ reaches the smallest peak rate and exhibits a fairly concentric shape around the adaptation target position. **(E)** The visual pre-saccadic target ${\vec{V}}_{1}$, the motor command $\vec{M}$ and the internal saccade estimate ${\vec{CD}}_{V}$ adapt to minimize the postdictive motor error $\vec{E}$. The shaded, blue area in the background are the measured saccade vectors ± SE. Further depicted signals are the postdicted pre-saccadic target ${\vec{\hat{V}}}_{1}$, the predicted post-saccadic target ${\vec{\hat{V}}}_{2}$ and the visual post-saccadic target ${\vec{V}}_{2}$. **(F)** The visual, motor and ${CD}_{V}$ adaptation fields and the post-saccadic localization exhibit a scaled, radial and asymmetric shape. The shaded areas depict the data and the arrows depict the model fit.

Fig 6 presents the model fit to the subjects’ mean data across the 200 adaptation saccades and the pre- and post-adaptation state of ${\vec{V}}_{1}$ (fitted to pre-saccadic localizations), ${\vec{\hat{V}}}_{2}$ (fitted to post-saccadic localizations with respect to saccade landing) and $\vec{M}$ (fitted to saccade vectors) across the 11 probe positions. Table 1 provides all fitted parameters.

**Table 1:** Fitted parameters of the model.

Description	Visual	Motor	CD
Gain fields $\omega_{v}$, $\omega_{m}$, $\omega_{cd}$:			
Constants in the pre-adaptation state	$\omega_{v_{C}}$ = 0.978	$\omega_{m_{C}}$ = 0.962	$\omega_{{cd}_{C}}$ = 1.020
Learning distributions $\alpha_{v}$, $\alpha_{m}$, $\alpha_{cd}$:			
Scaling factors	$\phi_{v}$ = 0.005	$\phi_{m}$ = 0.008	$\phi_{cd}$ = -0.003
Foveal width	$\sigma_{v_{F}}$ = 0.55	$\sigma_{m_{F}}$ = 0.48	$\sigma_{{cd}_{F}}$ = 1.10
Peripheral width	$\sigma_{v_{P}}$ = 2.01	$\sigma_{m_{P}}$ = 2.66	$\sigma_{{cd}_{P}}$ = 1.18
Orthogonal width	$\sigma_{v_{O}}$ = 1.04	$\sigma_{m_{O}}$ = 1.06	$\sigma_{{cd}_{O}}$ = 1.13

Here we report the fitted parameters of the model. The gain field constants $\omega_{v_{C}}$, $\omega_{m_{C}}$ and $\omega_{{cd}_{C}}$ were fitted to the pre-adaptation state. The parameters of the learning distributions were fitted to the trial-by-trial saccade adaptation and the post-adaptation state. The foveal, peripheral and orthogonal widths of the learning distributions are defined in dva. The learning distributions were restricted to extend maximally into the fovea with the constraints $\sigma_{v_{F}}\leq \frac{1}{3}\delta_{1}$, $\sigma_{m_{F}}\leq \frac{1}{3}\delta_{1}$ and $\sigma_{{cd}_{F}}\leq \frac{1}{3}\delta_{1}$ with $\delta_{1}$ being target eccentricity. The adaptation drop factor κ = 0.334 was fitted to capture the percentage of adaptation decline from the end of the adaptation phase to the post-adaptation phase. Fixed parameters were the pre-saccadic target ${\vec{P}}_{1}{\;}^{\left(n\right)}=\left(\begin{array}{c} 12\\ 0 \end{array}\right)$ and the peri-saccadic target step ${\vec{P}}_{s}{\;}^{\left(n\right)}=\left(\begin{array}{c} -3\\ 0 \end{array}\right)$ in saccade adaptation trials.

Fig 6A shows the gain fields $\omega_{v}$, $\omega_{m}$ and $\omega_{cd}$ in the pre-adaptation state (left column) that we fitted each as a uniform, horizontal plane defined by the fitted constants $\omega_{v_{C}}$ = 0.978, $\omega_{m_{C}}$ = 0.962 and $\omega_{{cd}_{C}}$ = 1.020 (Fig 6A). Hence, the gain fields were fairly well calibrated in the pre-adaptation state, but, with $\omega_{v_{C}}$ and $\omega_{m_{C}}$ being slightly < 1, the visual and motor gain fields reflect the slight underestimation of pre-saccadic target eccentricity as well as the saccade undershoot in the pre-adaptation state (Fig 6E). The post-saccadic target is predicted to appear in the fovea ($r_{{\hat{V}}_{2}}$ in Fig 6B, left column). However, due to the intra-saccadic inward target step, the target actually appears on the left horizontal meridian with respect to the fovea after saccade offset ($r_{V_{2}}$ in Fig 6C, left column). Thus, the postdictive motor error ($\vec{E}$ in Fig 6E) teaches the gain fields $\omega_{v}$, $\omega_{m}$ and $\omega_{cd}$ to adapt for error minimization.

Fig 6D depicts the fitted learning distributions $\alpha_{v}$, $\alpha_{m}$ and $\alpha_{cd}$ that define how much is learnt from error on a given trial at each position in the field. The motor learning distribution $\alpha_{m}$ exhibits the highest peak rate of $\phi_{m}$ = 0.008 at the adaptation target location compared to the visual and CD learning distributions with the fitted peak rates $\phi_{v}$ = 0.005 and $\phi_{cd}$ = -0.003. The CD learning distribution is negatively polarized as the forward dynamics model is the counterpart of the inverse model. Moreover, the motor learning distribution $\alpha_{m}$ has the widest spatial expansion compared to the visual and the CD learning distributions $\alpha_{v}$ and $\alpha_{cd}$. To account for the asymmetry of the adaptation fields along the amplitude dimension, we fitted the Gaussian widths of the learning distributions independently for the foveal and the peripheral amplitude direction with respect to the adaptation target, i.e. separately for decreasing vs. increasing amplitudes. The motor and visual learning distributions $\alpha_{m}$ and $\alpha_{v}$ exhibit an asymmetric shape along the amplitude axis. $\alpha_{m}$ extends 1.44 dva into the foveal amplitude dimension ($\sigma_{m_{F}}$ = 0.48 dva) and 7.88 dva into the peripheral amplitude dimension ($\sigma_{m_{P}}$ = 2.66 dva). $\alpha_{v}$ is smaller than $\alpha_{m}$ but also shows asymmetry with 1.65 dva expansion into the foveal amplitude dimension ($\sigma_{v_{F}}$ = 0.55 dva) and 6.03 dva expansion into the peripheral amplitude dimension ($\sigma_{v_{P}}$ = 2.01 dva). However, the asymmetry is more pronounced in $\alpha_{m}$ than in $\alpha_{v}$. In the orthogonal amplitude dimension (that was fitted with one parameter for both directions) $\alpha_{v}$ and $\alpha_{m}$ have almost the same widths ($\sigma_{v_{O}}$ = 1.04, i.e. 3.12 dva expansion in each direction; $\sigma_{m_{O}}$ = 1.06, i.e. 3.18 dva expansion in each direction). By contrast, the CD learning distribution $\alpha_{cd}$ does not follow the asymmetry but exhibits a fairly concentric shape with 3.30 dva expansion in the foveal amplitude dimension ($\sigma_{{cd}_{F}}$ = 1.10 dva), 3.54 dva expansion into the peripheral amplitude dimension ($\sigma_{{cd}_{P}}$ = 1.18 dva) and 3.39 dva into each direction of the orthogonal amplitude dimension ($\sigma_{{cd}_{O}}$ = 1.13 dva).

Fig 6F shows the adaptation fields, i.e. the change of ${\vec{V}}_{1}$, $\vec{M}$, ${\vec{CD}}_{V}$ and of the post-saccadic localization of the target in the respective experimental task. The shades in the background are the data and the arrows in front are the model fit. The ${\vec{CD}}_{V}$ change is larger than the ${\vec{V}}_{1}$ change as ${\vec{CD}}_{V}$ is a result of the large $\vec{M}$ change and the respective adaptation of $\omega_{cd}$ in the opposite direction.

When the motor command $\vec{M}$ converges to a new steady state at the end of the adaptation phase, the postdictive motor error $\vec{E}$ is almost nullified (Fig 6E). Consistent with Masselink & Lappe (2021) [14] and Masselink *et al*. (2023) [15] ${\vec{CD}}_{V}$ monitors the actual saccade change during adaptation but underestimates its size.

To account for the drop of the adaptation level that is usually observed between the end of the adaptation phase and the subsequently measured after-effect in the post-adaptation phase, we fitted the adaptation drop factor κ = 0.334. Hence, in the post-adaptation phase, the gain fields $\omega_{v}$, $\omega_{m}$ and $\omega_{cd}$ decayed by 33.4% towards the pre-adaptation state.

For goodness of fit, we obtained the residual standard errors *RSE*_*pre*,*post*_ = 0.52 dva (pre- and post-adaptation phase across horizontal and vertical saccade vectors, pre- and post-saccadic localizations at the 11 probe positions) and *RSE*_*adapt*_ = 0.23 dva (adaptation phase across horizontal and vertical saccade vectors at the adaptation target position).

In sum, the distribution of learning differs between gain field maps. Motor learning of $\omega_{m}$ exhibits the highest peak rate and covers the largest spatial area with a span of 9.32 dva along the amplitude dimension and a pronounced asymmetry of 1:5.5, i.e. it largely expands into the periphery. Visual learning of $\omega_{v}$ exhibits a medium sized peak rate, spans 7.68 dva along the amplitude dimension and shows an asymmetry of 1:3.7 in favor of the periphery. Learning of $\omega_{cd}$ occurs with a smaller peak rate in a fairly concentric area of 6.84 dva width along the amplitude dimension. Along the orthogonal amplitude dimension, the widths of the learning distributions are very similar.

## Discussion

We have presented a model that explains adaptation of saccade amplitudes in any saccade direction based on population-coded learning across visual, motor and CD gain field maps. In the model, learning acts via a polar-angle encoded error on the two-dimensional population codes for visual target localization, saccade amplitude and the internal representation of the saccade based on corollary discharge (CD). We fitted the model to saccade amplitudes and pre- and trans-saccadic visual localizations for different target positions before, during and after adaptation to a peri-saccadic inward target step. We show that the spatial layout of the adaptation field is explained by three mechanisms: 1) its scaled locality is explained by population coding, 2) its radial shape is explained by learning operating in polar-angle coordinates, and 3) its asymmetry is explained by an asymmetric shape of learning rates along the amplitude dimension. Learning of the saccadic population code exhibits the highest peak rate, the broadest extension along the amplitude dimension and the most pronounced asymmetry in favor of the spatial periphery, while for the visual and the internal saccade representation, learning appears more localized. Our findings support the idea that learning acts on the level of a neural population that collectively encodes the amplitude and direction of a saccade. Moreover, our results suggest that not only the representation of the visual target position and the saccade command but also the internal representation of saccade size has a response field that monitors only part of the ongoing saccade changes during learning. Our model can be used to study oculomotor learning in multiple contexts, e.g. how visuomotor behavior is shaped by spatial generalization or interference of learning at different sites in the visual field.

### Learning operates on gain field maps for visual, motor and internal saccade representation

The gain field model implements visuomotor plasticity by adaptive visual, motor and CD gain field maps that scale the response distribution and can learn from error.

Learning of the motor gain field covers the greatest spatial area, exhibits the highest peak rate at the adaptation target position and a pronounced asymmetry along the amplitude dimension, i.e. it expands far into the periphery. The size and peakedness of the motor learning distribution (as compared to the visual and the CD learning distribution) means that most part of the error is credited to a failure of the saccadic motor command. On neural level, this means that neurons that are tuned to saccades of intermediate distance to the adaptation target partially adapt, too. Thereby, the speed of saccade changes is driven not only by the high peak rate but also by the width of the distribution, since the activities in the vicinity contribute to the population vector that is computed across the motor population response. The asymmetry of the learning distribution in favor of the periphery is consistent with experimental results of Collins *et al*. (2007) [25] and Schnier *et al*. (2010) [22].

Learning of the visual gain field means that part of the error is assigned to an internal failure of visual target representation. Changes of visual target localization at the adaptation target position are usually small but consistent [14, 22, 25, 27, 28, 52, 53], as is its transfer to surrounding positions [22, 25]. This is confirmed by our results in that changes of visual target localization are smaller than saccade changes, and the visual learning distribution is notably narrower than the motor learning distribution. In the model fit, the visual learning distribution showed asymmetry in that it expands wider into the peripheral than into the foveal direction. Yet, due to the small effect, we are cautious in interpreting this asymmetry. Thus, it would be interesting to further examine the shape of the visual learning distribution with a larger intra-saccadic target step and more transfer positions.

Learning of the CD gain field captures how well the forward dynamics model tracks the ongoing motor changes during learning. Consistent with previous results, we found that the internal saccade estimate reflects most of the saccade changes but still underestimates its amount, i.e. it decouples from the actual saccade size [14, 15, 54–56]. This underestimation was transferred to saccades to neighboring targets, i.e. the ${CD}_{V}$ adaptation field was less profound than the saccade adaptation field. Accordingly, the CD learning distribution was confined to a more local area as compared to the motor learning distribution, and appeared rather concentric around the adaptation target position. Yet, further studies are needed to draw secure conclusions on the shape of CD learning across the visual field.

### A plastic, population-coded internal representation of the saccade

Our approach to quantify the visuomotor system’s internal estimate of how large the saccade will be (referred to as the ${CD}_{V}$ signal) is based on the comparison between the localization of a briefly flashed stimulus with vs. without performing a saccade in between. Hence, after a saccade that lands without any visual references, subjects need to rely on their internal estimate of saccade size in order to localize, in post-saccadic coordinates, the position of the flash that was presented in pre-saccadic coordinates. A mismatch between these judgements reveals a mismatch between the actual saccade and its internal representation, that, according to our data, arises in the course of learning. These results are consistent with previous studies that have demonstrated changes in post-saccadic localization at the adaptation target position [22, 25, 55, 57–59] and at transfer positions [22, 25]. Yet, in Collins *et al*. (2007) [25] and Schnier *et al*. (2010) [22], subjects localized a flash presented at the transfer position after executing a saccade to the adaptation target (and not to the transfer position, as in our paradigm). Hence, the results of Collins *et al*. (2007) [25] and Schnier *et al*. (2010) [22] did not allow to draw conclusions on the internal estimate of saccades to targets other than the adaptation target. As far as we know, our study is the first that demonstrates a transfer of ${CD}_{V}$ changes to other saccades, i.e. that the internal saccade representation has an adaptation field.

The plasticity of the internal saccade estimate ${CD}_{V}$ and its transfer to nearby targets is at odds with the assumption that ${CD}_{V}$ must always indicate the correct saccade vector. In our view, this assumption is fed by the logic that a copy of the motor command, i.e. the efference copy or corollary discharge [60–62], cannot deviate from the actual motor command, i.e. it cannot be wrong. We agree, yet, a copy of the motor command is in motor coordinates and hence, it cannot, by itself, indicate the saccade size in visuospatial coordinates. For this transformation a forward dynamics model is needed that should be adaptive with respect to the dynamics of the eye muscles, i.e. to whether the eye muscles are fatigued or strong [1, 54, 63–65]. Hence, the accuracy of the internal saccade estimate ${CD}_{V}$ depends on the calibration of the forward dynamics model. That the internal saccade estimate can deviate from the actual oculomotor behavior is acknowledged for pursuit eye movements [66, 67] and has been shown for saccades when the SC-MD-FEF pathway for CD transmission is lesioned [68] or experimentally perturbed [69–72]. Moreover, Cavanaugh *et al*. (2016) [72] demonstrated that inactivation of MD thalamus in the macaque monkey causes a bias in trans-saccadic perception, similar to our finding after adaptation of saccadic eye movements.

In our model, the plasticity of the forward dynamics model is implemented as plasticity of a CD gain field map. This follows the idea of a population code representation for ${CD}_{V}$ that explains why we find a ${CD}_{V}$ adaptation field, i.e. why adaptation of the internal saccade estimate transfers to saccades in the vicinity. It seems worthwhile to test this idea. At least, population coding mechanisms are known from areas that transmit the corollary discharge signal, like the SC [19, 20] and the FEF [31–33].

### The scaled, radial and asymmetric shape of the adaptation field

Our data confirmed four essential properties of the adaptation field. First, the adaptation field is scaled, i.e. it is locally limited with a peak at the adaptation target position and a gradual decline to the outside [16, 18, 22]. Second, the adaptation field is radial, i.e. the transfer of adaptation is oblique on both sides of the amplitude axis (and not parallel to the amplitude axis; [7, 17, 23]). Third, the adaptation field, at least for the saccade domain, is asymmetric along the amplitude dimension with more transfer to larger saccade amplitudes than to shorter saccade amplitudes [7, 17, 23]. Forth, saccade changes are accompanied by changes in pre- and trans-saccadic target localization that show an adaptation field as well [22, 25].

In accordance with our model that mirrors these characteristics, the local scaling of adaptation fields can be explained if learning does not act on the population vector but already on the level of the neuronal population that collectively encodes saccade amplitude and direction (for polar-angle encoding see [49, 50]). Moreover, the radial shape of the adaptation field can be explained if learning takes place upstream from the component decomposition of the saccade [7, 17, 23, 73], i.e. in polar-angle coordinates. In our model, this is reflected by the polar-angle encoding of error that teaches the saccade amplitude to adjust instead of teaching its separate horizontal and vertical components. By contrast, if learning acted on the horizontal and vertical saccade components, the adaptation field should be parallel, and not oblique, to the amplitude axis. In our model, the asymmetry of the adaptation field is explained by higher learning rates in the visual periphery than in more foveal-directed positions. This could also be caused by non-linear representations of saccade amplitudes, like along the rostral-caudal axis of the SC where small amplitude saccades are over-represented compared to large amplitude saccades [19]. Thus, a high number of neurons that are tuned to small saccades could absorb part of the adaptive effect, leaving small amplitude saccades less affected by adaptation at a medium-sized target position.

### Neurophysiological sites of population-coded learning

Although our model is not intended as a detailed model of the physiology of saccadic adaptation and contains a number of simplifications of the physiological details it can nevertheless provide some conceptual indications regarding possible sites of population-coded learning. Whether adaptation alters activity in the polar-angle eye movement map of the intermediate SC [19, 20] remains a matter of debate. Several studies did not find a change in the locus of SC activity during adaptation [74–76]. However, an increase in discharge rate has been reported before the onset of adapted saccades in the SC [77] and along one of its descending routes, i.e. in the nucleus reticularis tegmenti pontis (NRTP) [78] and in the caudal fastigial nucleus (cFN) [79, 80]. The cFN relays the output of the cerebellar oculomotor vermis which is highly associated with error encoding and motor adjustments [63, 81–83] and has been shown to hold retinotopic properties [36]. The Purkinje cells in the oculomotor vermis are sensitive to saccade direction and encode saccade amplitude. Yet, saccade amplitude is rather encoded by the duration of the population activity than by spatial tuning [84, 85]. Moreover, the cerebellum holds interconnections with a variety of cortical areas, like the FEF and parietal cortex [86–88]. These areas are involved in adaptation [26, 89], show retinotopic organization [90–92] and population coding [31, 33–35]. Hence, it is possible that these areas contribute to plasticity of visual and motor representations and of the internal saccade representation during learning. A potential population code plasticity for the internal representation of the saccade could be implemented along several routes that are known for corollary discharge transmission, like from SC via MD thalamus to the FEF [29, 93, 94], from SC through the thalamic pulvinar to parietal and occipital cortex [95, 96], from the cerebellum through VL thalamus to frontal cortex [97–99] and from FEF via the basal ganglia to SC [64, 100].

### Learning from postdictive motor error

In our model, gain field changes were driven by postdictive motor error. This means that the oculomotor system derives a postdictive update of the pre-saccadic target based on post-saccadic visual input and calculates the error of the motor command with respect to this position. Minimization of postdictive motor error well explains the simultaneous changes of saccade amplitude, pre- and post-saccadic localization [14, 15, 101] as well as its transfer to nearby spatial positions, as shown in the present data. Most adaptation models explain saccade changes only and use visual prediction error as the driving force of learning [9, 12]. In these models, the prediction is usually quantified as the fixed saccade undershoot that was measured in the baseline, even if the saccade vector changes during learning. However, taking the spatial gap that opens up between pre- and post-saccadic localization into account, i.e. the bias in the internal representation of the saccade, it becomes clear that visual prediction error is not minimized during learning [14]. By contrast, learning from postdictive motor error well explains changes of visual, motor and internal saccade representations and is compatible with saccadic suppression of displacement [102, 103] in that the world is assumed stable across saccades.

### Model choices and limitations

Our model does not reflect a detailed neural representation of all stages of the oculomotor transformation but is a conceptual model that explains how adaptation is transferred to neighboring positions across visual, motor and internal saccade representations. Hence, our modeling approach adopts a set of simplifications in representing sensorimotor signals to focus on the examination of the spatial shape of the learning transfer. We choose an exclusively eye-centered reference frame, as it is widely represented in areas such as the SC and FEF [41, 104]. We model response fields as Gaussian [19, 105], though we acknowledge that motor response fields yield more complex and asymmetric profiles [106, 107]. We implement spatial updating through a simple shift of the population response by the internal estimate of saccade size, simplifying the more complex neural processes involved in updating spatial representations following eye movements [38–41]. Our model of the forward dynamics process is also kept deliberately simple without referring to the detailed physiology, for example in the cerebellum [108, 109]. These choices serve to prioritize an interpretable isolation of how learning is generalized across visual, motor and internal saccade maps.

### Spatial generalization and interference of learning at different sites in the visual field

We have used our gain field adaptation model to explain spatial transfer of learning, i.e. how learning at a specific target position transfers to saccades of different amplitude and direction. Beyond that, our model can be used to explore various spatial dependencies of learning. For example, in position-dependent adaptation, the saccade target may step inward in trials to a specific primary target position, and step outward in trials to a different primary target position. It has been shown that the oculomotor system is able to learn such conflicting tasks demands [110–112]. However, it remains to be examined how these learning processes interfere with each other, and how this depends on the spatial distance of the two targets. In addition, learning the same task demand at different primary target positions may lead to spatial generalization of learning. Rolfs *et al*. (2010) [113] showed that learning from inward target steps has roughly the same or even a slighly higher speed when performed across saccades in random directions (referred to as global adaptation) as when the saccades were always performed in the same direction. Our model could help to dissociate how much global adaptation is boosted by spatial transfer, i.e. how much a saccade benefits from learning at neighboring positions in previous trials, and how much of the global adaptation speed must result from a different process that, e.g., could be driven by the error regularity across saccade directions. Moreover, our model could unravel how global adaptation is accompanied by changes in visual target and internal saccade representation. Beyond that, sequential learning of different task demands (e.g. learning from inward errors in a first block followed by outward errors in the next block) may leave footprints in the population code that could manifest itself in the temporal domain and hence, explain phenomena of motor memory [114]. These paradigms could be used to further validate our model, that could, in turn, provide insights into the underyling mechanisms.

### Interaction with direction adaptation

In future work, it would be interesting to extend the model to account for error direction in addition to error amplitude. This could be achieved by introducing an additional direction gain field for the visual, motor, and CD domains, allowing the model to adapt not only the length of the population vector but also its direction. Such an extension would enable the model to simulate adaptation to directional target jumps and make predictions about how saccade direction adaptation unfolds over time. Moreover, this framework could be used to investigate potential interactions between amplitude and direction adaptation, particularly in cases where the target jump has both an amplitude and a directional component. Examining whether these two forms of adaptation operate independently or affect each other could provide valuable insights into the neural mechanisms underlying saccade learning.

### From a 1D to a 2D perspective in modeling oculomotor learning

The brain represents the visual world and our movement goals by spatially tuned population codes in two-dimensional topographic maps. However, despite the shape of adaptation transfer to nearby spatial positions, computational models of saccadic adaptation have largely remained grounded in the classical perspective of one-dimensional movement vector adjustments. We think that oculomotor learning should be studied in a framework that accounts for the two-dimensionality of visuomotor representations, in order to capture how it shapes adaptation phenomena, like spatial transfer, spatial interference of learning, or motor memory.

## Methods

### Participants

Data were recorded from *N* = 13 healthy subjects (26.4 ± 7.8 years, 5 female, 8 male) with normal or corrected-to-normal vision who were naïve to the objectives of the experiment.

### Ethics statement

All subjects gave written informed consent prior to the first recording session. The experiment followed the 2008 Declaration of Helsinki and was approved by the Ethics Committee of Department 7 (Psychology and Sport Science) of the University of Münster with protocol number 2015-21-ML.

### Model

The model represents each signal by a population response across the two-dimensional visual field. Locations are encoded by population vectors that are calculated across the population response described with the help of the two-dimensional Gaussian distribution:

$$f^{\left(\vec{p};\vec{\mu },\vec{\sigma }\right)}=\exp\left(-\left(\frac{(p_{x}-\mu_{x}{)}^{2}}{2\sigma_{x}^{2}}+\frac{(p_{y}-\mu_{y}{)}^{2}}{2\sigma_{y}^{2}}\right)\right)$$ (1)

where $\vec{p}=\left(\begin{array}{c} p_{x}\\ p_{y} \end{array}\right)$ is the retino-centric position, $\vec{\mu }=\left(\begin{array}{c} \mu_{x}\\ \mu_{y} \end{array}\right)$ is the center and $\vec{\sigma }=\left(\begin{array}{c} \sigma_{x}\\ \sigma_{y} \end{array}\right)$ is the standard deviation of the Gaussian.

**Pre-saccadic computations.** The population response $r_{I_{1}}{\;}^{\left(\vec{p},n\right)}$ to the pre-saccadic target ${\vec{P}}_{1}$ on the input map for trial number *n* is (Fig 1a)

$$r_{I_{1}}{\;}^{\left(\vec{p},n\right)}=\frac{f^{\left(\vec{p};{\vec{P}}_{1},{\vec{\sigma }}_{1}\right)}}{\sum_{\vec{p}}f^{\left(\vec{p};{\vec{P}}_{1},{\vec{\sigma }}_{1}\right)}}$$ (2)

with $\sigma_{1x}=\sigma_{1y}=\frac{1}{3}\delta_{1}$ where $\delta_{1}=|{\vec{P}}_{1}|$, i.e. target eccentricity. Hence, the width and the height of the population response linearly depends on target eccentricity such that more peripheral targets produce a broader but flatter population response. Moreover, the population response has its limits in the fovea.

The visual gain field $\omega_{v}{\;}^{\left(\vec{p},n\right)}$ routes the activity on the input map onto a visual map, resulting in the visual population response $r_{V_{1}}{\;}^{\left(\vec{p},n\right)}$ (Fig 1b):

$$r_{V_{1}}{\;}^{\left(\vec{p},n\right)}=r_{I_{1}}{\;}^{\left(\vec{p},n\right)}\;\omega_{v}{\;}^{\left(\vec{p},n\right)}$$ (3)

The visual pre-saccadic target position is read out from the visual map by:

$${\vec{V}}_{1}{\;}^{\left(n\right)}=\sum_{\vec{p}}r_{V_{1}}{\;}^{\left(\vec{p},n\right)}\;\vec{p}$$ (4)

Hence, if the whole visual gain field $\omega_{v}{\;}^{\left(\vec{p},n\right)}$ is equal to 1, the visual pre-saccadic target will be accurately localized on the visual map.

The visual population response is scaled by the motor gain field $\omega_{m}{\;}^{\left(\vec{p},n\right)}$ to transform the visual map into a motor map (Fig 1c):

$$r_{M}{\;}^{\left(\vec{p},n\right)}=r_{V_{1}}{\;}^{\left(\vec{p},n\right)}\;\omega_{m}{\;}^{\left(\vec{p},n\right)}$$ (5)

The motor population response $r_{M}{\;}^{\left(\vec{p},n\right)}$ determines the motor command:

$$\vec{M}{\;}^{\left(n\right)}=\sum_{\vec{p}}r_{M}{\;}^{\left(\vec{p},n\right)}\;\vec{p}$$ (6)

Hence, the motor gain field $\omega_{m}{\;}^{\left(\vec{p},n\right)}$ acts as an inverse model that transforms a visual representation into a motor representation. If the motor gain field is perfectly tuned, the whole motor gain field will be equal to 1 such that the saccade will land on the visual pre-saccadic target location.

The motor population response is scaled by the CD gain field $\omega_{cd}{\;}^{\left(\vec{p},n\right)}$ to transform the motor map into a ${CD}_{V}$ map (Fig 1d):

$$r_{CD_{V}}{\;}^{\left(\vec{p},n\right)}=r_{M}{\;}^{\left(\vec{p},n\right)}\;\omega_{cd}{\;}^{\left(\vec{p},n\right)}$$ (7)

Hence, the CD gain field $\omega_{cd}{\;}^{\left(\vec{p},n\right)}$ acts as a forward dynamics model that re-transforms motor coordinates back into visuospatial coordinates. The population vector across the ${CD}_{V}$ map determines the computed displacement of visual space:

$${\vec{CD}}_{V}{\;}^{\left(n\right)}=\sum_{\vec{p}}r_{CD_{V}}{\;}^{\left(\vec{p},n\right)}\;\vec{p}$$ (8)

If the whole CD gain field $\omega_{cd}{\;}^{\left(\vec{p},n\right)}$ is equal to 1, it is perfectly tuned such that ${\vec{CD}}_{V}{\;}^{\left(n\right)}$ matches the actual saccade size.

The population response $r_{{\hat{V}}_{2}}{\;}^{\left(\vec{p},n\right)}$ on the prediction map (Fig 1e) is:

$$r_{{\hat{V}}_{2}}{\;}^{\left(\vec{p},n\right)}=r_{V_{1}}{\;}^{\left(\vec{p}+{\vec{CD}}_{V}{\;}^{\left(n\right)},n\right)}$$ (9)

Hence, the post-saccadic target is predicted to appear at the position:

$${\vec{\hat{V}}}_{2}{\;}^{\left(n\right)}=\sum_{\vec{p}}r_{{\hat{V}}_{2}}{\;}^{\left(\vec{p},n\right)}\;\vec{p}$$ (10)

**Saccade execution.** The saccade is executed with the motor noise ${\vec{\epsilon }}_{M}{\;}^{\left(n\right)}$, resulting in the saccade vector:

$${\vec{P}}_{M}{\;}^{\left(n\right)}={\vec{M}}^{\left(n\right)}+{\vec{\epsilon }}_{M}{\;}^{\left(n\right)}$$ (11)

Due to peri-saccadic target step ${\vec{P}}_{s}{\;}^{\left(n\right)}$ and motor error ${\vec{\epsilon }}_{M}{\;}^{\left(n\right)}$, the oculomotor system experiences the physical disruption ${\vec{P}}_{d}{\;}^{\left(n\right)}$ across the saccade:

$${\vec{P}}_{d}{\;}^{\left(n\right)}={\vec{P}}_{s}{\;}^{\left(n\right)}-{\vec{\epsilon }}_{M}{\;}^{\left(n\right)}$$ (12)

With respect to saccade landing, the post-saccadic target is placed at the position:

$${\vec{P}}_{2}{\;}^{\left(n\right)}={\vec{P}}_{1}{\;}^{\left(n\right)}+{\vec{P}}_{d}{\;}^{\left(n\right)}-{\vec{P}}_{M}{\;}^{\left(n\right)}$$ (13)

**Post-saccadic computations.** The population response $r_{I_{2}}{\;}^{\left(\vec{p},n\right)}$ to the post-saccadic target on the input map (Fig 1f) is

$$r_{I_{2}}{\;}^{\left(\vec{p},n\right)}=\frac{f^{\left(\vec{p};{\vec{P}}_{2},{\vec{\sigma }}_{2}\right)}}{\sum_{\vec{p}}f^{\left(\vec{p};{\vec{P}}_{2},{\vec{\sigma }}_{2}\right)}}$$ (14)

with $\sigma_{2x}=\sigma_{2y}=\frac{1}{3}\delta_{2}$ where $\delta_{2}=|{\vec{P}}_{2}|$, i.e. post-saccadic target eccentricity. To restrict the height and narrowness of the population response for foveal targets, we set $\sigma_{2x}\geq$ 0.5 and $\sigma_{2y}\geq$ 0.5.

The visual population response $r_{V_{2}}{\;}^{\left(\vec{p},n\right)}$ (Fig 1g) to the post-saccadic target on the visual map is

$$r_{V_{2}}{\;}^{\left(\vec{p},n\right)}=r_{I_{2}}{\;}^{\left(\vec{p},n\right)}\;\omega_{v}{\;}^{\left(\vec{p},n\right)}$$ (15)

resulting in the visual post-saccadic target position:

$${\vec{V}}_{2}{\;}^{\left(n\right)}=\sum_{\vec{p}}r_{V_{2}}{\;}^{\left(\vec{p},n\right)}\;\vec{p}$$ (16)

The population response $r_{{\hat{V}}_{1}}{\;}^{\left(\vec{p},n\right)}$ on the postdiction map (Fig 1h) results from a backward coordinate shift of $r_{V_{2}}{\;}^{\left(\vec{p},n\right)}$ by ${\vec{CD}}_{V}{\;}^{\left(n\right)}$:

$$r_{{\hat{V}}_{1}}{\;}^{\left(\vec{p},n\right)}=r_{V_{2}}{\;}^{\left(\vec{p}-{\vec{CD}}_{V}{\;}^{\left(n\right)},n\right)}$$ (17)

The postdicted target position is:

$${\vec{\hat{V}}}_{1}{\;}^{\left(n\right)}=\sum_{\vec{p}}r_{{\hat{V}}_{1}}{\;}^{\left(\vec{p},n\right)}\;\vec{p}$$ (18)

**Adaptation of visual, motor and CD gain field maps.** The postdictive motor error $\vec{E}{\;}^{\left(n\right)}$ (Fig 1i) is computed as the error of the motor command ${\vec{M}}^{\left(n\right)}$ with respect to the postdicted target position ${\vec{\hat{V}}}_{1}{\;}^{\left(n\right)}$:

$$\vec{E}{\;}^{\left(n\right)}={\vec{\hat{V}}}_{1}{\;}^{\left(n\right)}-{\vec{M}}^{\left(n\right)}$$ (19)

For adaptation, the postdictive motor error is encoded as a directed amplitude error:

For $P_{1x}{\;}^{\left(n\right)}$ ≠ 0:

$$\delta_{E}{\;}^{\left(n\right)}=\frac{E_{x}{\;}^{\left(n\right)}\;P_{1x}{\;}^{\left(n\right)}}{\left|E_{x}{\;}^{\left(n\right)}\;P_{1x}{\;}^{\left(n\right)}\right|}\;|\vec{E}{\;}^{\left(n\right)}|$$ (20)

For $P_{1x}{\;}^{\left(n\right)}$ = 0:

$$\delta_{E}{\;}^{\left(n\right)}=\frac{E_{y}{\;}^{\left(n\right)}\;P_{1y}{\;}^{\left(n\right)}}{\left|E_{y}{\;}^{\left(n\right)}\;P_{1y}{\;}^{\left(n\right)}\right|}\;|\vec{E}{\;}^{\left(n\right)}|$$ (21)

Thereby, $\left|\vec{E}{\;}^{\left(n\right)}\right|$ is the magnitude of the error, and $\frac{E_{x}{\;}^{\left(n\right)}\;P_{1x}{\;}^{\left(n\right)}}{\left|E_{x}{\;}^{\left(n\right)}\;P_{1x}{\;}^{\left(n\right)}\right|}$ directs the error such that $\delta_{E}{\;}^{\left(n\right)}$ is < 0 for inward target steps and $\delta_{E}{\;}^{\left(n\right)}$ is > 0 for outward target steps, independently whether adaptation takes place in the left hemifield ($P_{1x}{\;}^{\left(n\right)}<0$) or in the right hemifield ($P_{1x}{\;}^{\left(n\right)}>0$). The encoding of the postdictive motor error as a directed amplitude error causes the change in amplitude to be transferred as a whole to surrounding targets, rather than distributing the changes separately across the horizontal and vertical components.

Accordingly, the visual, motor and CD gain fields adapt to reduce postdictive motor error:

$$\omega_{v}{\;}^{\left(\vec{p},n+1\right)}=\omega_{v}{\;}^{\left(\vec{p},n\right)}+\alpha_{v}{\;}^{\left(\vec{p}\right)}\;\delta_{E}{\;}^{\left(n\right)}$$ (22)

$$\omega_{m}{\;}^{\left(\vec{p},n+1\right)}=\omega_{m}{\;}^{\left(\vec{p},n\right)}+\alpha_{m}{\;}^{\left(\vec{p}\right)}\;\delta_{E}{\;}^{\left(n\right)}$$ (23)

$$\omega_{cd}{\;}^{\left(\vec{p},n+1\right)}=\omega_{cd}{\;}^{\left(\vec{p},n\right)}+\alpha_{cd}{\;}^{\left(\vec{p}\right)}\;\delta_{E}{\;}^{\left(n\right)}$$ (24)

with the visual, motor and CD learning distributions:

$$\alpha_{v}{\;}^{\left(\vec{p}\right)}=\phi_{v}\frac{f^{\left(\vec{p};{\vec{P}}_{1},{\vec{\sigma }}_{v}\right)}}{\sum_{\vec{p}}f^{\left(\vec{p};{\vec{P}}_{1},{\vec{\sigma }}_{v}\right)}}$$ (25)

$$\alpha_{m}{\;}^{\left(\vec{p}\right)}=\phi_{m}\frac{f^{\left(\vec{p};{\vec{P}}_{1},{\vec{\sigma }}_{m}\right)}}{\sum_{\vec{p}}f^{\left(\vec{p};{\vec{P}}_{1},{\vec{\sigma }}_{m}\right)}}$$ (26)

$$\alpha_{cd}{\;}^{\left(\vec{p}\right)}=\phi_{cd}\frac{f^{\left(\vec{p};{\vec{P}}_{1},{\vec{\sigma }}_{cd}\right)}}{\sum_{\vec{p}}f^{\left(\vec{p};{\vec{P}}_{1},{\vec{\sigma }}_{cd}\right)}}$$ (27)

The learning distributions specify the learning rate for each position across the visual, motor and CD gain fields. Hence, the learning distributions determine how much the gain fields learn from error in a given trial. Thereby, $\phi_{v}$, $\phi_{m}$ and $\phi_{cd}$ act as a scaling factor for the learning distribution. To take account for the asymmetry of the adaptation field along the amplitude dimension, we allowed the learning distributions to be differently shaped inward compared to outward of the adaptation target, with ${\vec{\sigma }}_{v}=\left(\begin{array}{c} \sigma_{v_{F}}\\ \sigma_{v_{O}} \end{array}\right)$, ${\vec{\sigma }}_{m}=\left(\begin{array}{c} \sigma_{m_{F}}\\ \sigma_{m_{O}} \end{array}\right)$ and ${\vec{\sigma }}_{cd}=\left(\begin{array}{c} \sigma_{{cd}_{F}}\\ \sigma_{{cd}_{O}} \end{array}\right)$ along the foveal amplitude dimension, and ${\vec{\sigma }}_{v}=\left(\begin{array}{c} \sigma_{v_{P}}\\ \sigma_{v_{O}} \end{array}\right)$, ${\vec{\sigma }}_{m}=\left(\begin{array}{c} \sigma_{m_{P}}\\ \sigma_{m_{O}} \end{array}\right)$ and ${\vec{\sigma }}_{cd}=\left(\begin{array}{c} \sigma_{{cd}_{P}}\\ \sigma_{{cd}_{O}} \end{array}\right)$ along the peripheral amplitude dimension (with ‘O’ indexing the orthogonal amplitude axis along which the learning distributions are symmetrically shaped). Learning distributions were first created with a Gaussian along the rightward horizontal axis and then rotated according to the target angle. Learning distribution were restricted to extend maximally into the fovea with the constraints $\sigma_{v_{F}}\leq \frac{1}{3}\delta_{1}$, $\sigma_{m_{F}}\leq \frac{1}{3}\delta_{1}$ and $\sigma_{{cd}_{F}}\leq \frac{1}{3}\delta_{1}$.

### Setup

The experiment was conducted in a dark room (luminance below $0.01\;{\text{cd/m}}^{2}$) with all sources of light removed. Subjects were seated with a chin rest and forehead support 62 cm in front of an Eizo FlexScan F930 monitor (Eizo, Hakusan, Japan; 40×30 cm, 1152×870 pixels, 32.8×25.8 dva, 75 Hz) that was covered with a dark foil to avoid visibility of the monitor background light. Subjects used a multi-touch trackpad (Apple Inc., Cupertino, CA) for localization judgements.

The right eye was recorded by an Eyelink 1000 at 1000 Hz (SR Research, Ontario, Canada) with a 1.5 dva position and a 22 $\frac{{\;}^{dva}}{s}$ velocity threshold for online detection of saccade onset. Saccade offset was detected online as soon as saccade velocity fell below 30 $\frac{{\;}^{dva}}{s}$. The experimental procedure was conducted by a Matlab script (Mathworks, Natick, MA).

### Tasks and procedure

Saccade adaptation was induced with a 12 dva rightward target that stepped 3 dva inward during saccade execution (Fig 4B). The pre- and post-adaptation phases tested the current state of saccade vector, pre-and post-saccadic localization for 11 probe positions including the 12 dva rightward adaptation target (Fig 4C). As the post-adaptation level could contain only a restricted amount of trials during which it is possible to maintain the adaptation level, every subject passed through the experimental session twice.

The probe targets were arranged on two invisible concentric circles around the adaptation target (on a 3 dva radius circle with 0, 90, 180 and 270 angular degree and on a 6 dva radius circle with 30, 90, 150, 210, 270 and 330 angular degree). Each trial started with a fixation dot placed 6 dva leftward of the screen center. The dot color revealed whether subjects had to perform a saccade (red for saccade trials and post-saccadic localization trials) or to keep fixation on the fixation point (green for pre-saccadic localization trials). If the subject had fixated the fixation dot with 1.5 dva maximum deviation for a random threshold duration between 500 and 1200 ms, the trial was initiated. Fixation dots and saccade targets measured 0.6 diameter.

**Saccade trials.** At fixation dot offset, a saccade target was displayed 12 dva to the right that, from the adaptation phase onwards, jumped 3 dva inward upon saccade onset (Fig 4B). In the pre-adaptation phase, the target remained at its initial position. After saccade offset, the target was visible for 500 ms.

**Post-saccadic localization trials.** At fixation dot offset, a saccade target was displayed at one of the 11 probe positions. After 40-50 ms, a white square of 0.6 dva width was flashed for 26.7 ms at a random position on an invisible circle of 1 dva radius around the probe position. Upon saccade onset, the target was erased. Subjects had to hold their gaze at the saccade landing position. A gaze deviation of 4 dva was accepted, otherwise a beep tone occurred until gaze position returned to the accepted fixation window. A grey dot cursor of 0.7 dva diameter turned on 500 ms after saccade offset at a random position within an invisible square of 16 dva side length around the adaptation target position. While keeping fixation at the saccade landing location, subjects had to localize the perceived flash position with the dot cursor. In case they had not perceived the flash, they were instructed to click at the lowest possible location, i.e. the invisible lower screen border.

**Pre-saccadic localization trials.** After fixation dot offset, subjects had to keep fixation at the invisible fixation dot location. After 40-50 ms, a white square was flashed. Subjects had to localize the perceived flash position with a dot cursor that appeared 770 ms after fixation dot offset while keeping fixation at the invisible fixation dot position. Parameters of the flashed square, dot cursor and fixation check procedure followed those of the post-saccadic localization trials.

Subjects practiced every trial type at the start of each session. The adaptation phase consisted of 200 saccade trials. The pre- and the post-adaptation phase contained a repeated sequence of saccade trial, post-saccadic localization trial and pre-saccadic localization trial. The saccade trials were always directed to the adaptation target with intra-saccadic target step in the post-adaptation phase to maintain the level of adaptation. The pre- and post-saccadic localization trials covered the 11 probe positions in random order (each position repeated 5 times, including the adaptation target position), resulting in 55 trials of each trial type, i.e. 3×55 trials = 165 trials in the pre- and in the post-adaptation phase. The inter-trial interval was 800 ms. In the middle of the pre- and of the post-adaptation phase as well as between phases, subjects took a self-paced break. Sessions took around 30 minutes each and were recorded at least 5 days apart.

### Data analysis

The data were analyzed offline in Matlab R2022a (Mathworks, Natick, MA). All reactive saccades with a latency of 100-400 ms that landed within ± 5 dva horizontally and vertically around the saccade target of the respective trial were accepted for analysis. A customized procedure with a combined velocity-acceleration criterion served for offline detection of saccade on- and offset. As the relation between post-saccadic target localization and saccade landing position is pivotal for a reliable ${\vec{CD}}_{V}$ estimate, in the pre- and in the post-adaptation phase only the saccades of the post-saccadic localization trials were used for further analysis.

Post-saccadic localizations without a valid primary saccade to the target were excluded. Localizations were accepted only if gaze was held successfully within ± 2 dva at the respective fixation position.

For each of the 11 probe positions, we calculated the median saccade vector ($\vec{M}$), median pre-saccadic localization ($\vec{V_{1}}$) and median post-saccadic localization (denoted as ${\vec{V}}_{2f}$, i.e. with respect to the fixation point) per subject before and after adaptation. On this basis, we derived the state of the computed displacement of visual space before and after adaptation (Fig 5C):

$${\vec{CD}}_{V}={\vec{V}}_{1}-{\vec{V}}_{2f}+\vec{M}$$ (28)

The data were averaged across the two sessions per subject, and averaged across subjects for the grand mean level. Two-sided one-sample t-tests or Wilcoxon signed rank tests were used in case normality distribution was violated. The significance level was 0.05.

### Model fitting

The model was fitted to the grand mean data with a visual field of ± 48 dva and 0.05 dva step width. The first and the last trial served as the pre- and the post-adaptation state. We fitted the gain field constants $\omega_{v_{C}}$, $\omega_{m_{C}}$ and $\omega_{{cd}_{C}}$ to the pre-adaptation state, and the learning distributions to the trial-by-trial saccade adaptation and the post-adaptation state. These were the scaling factors $\phi_{v}$, $\phi_{m}$ and $\phi_{cd}$, the width along the foveal amplitude dimension $\sigma_{v_{F}}$, $\sigma_{m_{F}}$ and $\sigma_{{cd}_{F}}$, along the peripheral amplitude dimension $\sigma_{v_{P}}$, $\sigma_{m_{P}}$ and $\sigma_{{cd}_{P}}$, and along the orthogonal amplitude dimension $\sigma_{v_{O}}$, $\sigma_{m_{O}}$ and $\sigma_{{cd}_{O}}$. To account for the drop of the adaptation level from the end of the adaptation phase to the adaptation after-effect in a post-adaptation phase, we fitted the adaptation drop factor κ that captures the percentage of gain field adaptation decay towards the pre-adaptation state. The fitting procedure minimized the weighted sum of squared errors *SSE*_*weighted*_. This included *SSE*_*pre*,*post*_ of the pre- and the post-adaptation phase (pre- and post-saccadic localizations as well as saccade vectors, i.e. 3 types × 2 time points (pre and post) × 2 dimensions (horizontal and vertical) × 11 target positions = 132 data points), and *SSE*_*adapt*_ of the adaptation phase (saccade vectors to the adaptation target, i.e. 200 trials × 2 dimensions (horizontal and vertical) = 400 data points). The visual pre-saccadic target position ${\vec{V}}_{1}$ was fitted to the pre-saccadic localizations, the motor command $\vec{M}$ was fitted to the saccade vectors and the predicted post-saccadic target position ${\vec{\hat{V}}}_{2}$ was fitted to the post-saccadic localizations with respect to the saccade landing location. Due to the unequal number of data points between phases, SSE minimization would favor a good fit of the adaptation phase (*q*_*adapt*_ = 400 data points) at the expense of the pre- and post-adaptation phase (*q*_*pre*,*post*_ = 132 data points). To ensure a balanced fit to all phases, we minimized the weighted sum of squared errors:

$$SSE_{weighted}=\eta_{pre,post}SSE_{pre,post}+\eta_{adapt}SSE_{adapt}$$ (29)

with the weights $\eta_{\text{pre,post}}=\frac{\left(q_{\text{adapt}}+q_{\text{pre,post}}\right)}{q_{\text{pre,post}}}\times$ 0.6 = 2.42 and $\eta_{\text{adapt}}=\frac{\left(q_{\text{adapt}}+q_{\text{pre,post}}\right)}{q_{\text{adapt}}}\times$ 0.4 = 0.53. Model fitting was performed with ${\vec{\epsilon }}_{M}{\;}^{\left(n\right)}=\left(\begin{array}{c} 0\\ 0 \end{array}\right)$. For goodness of fit, we calculated the residual standard errors:

$$RSE_{pre,post}=\sqrt{\frac{SSE_{pre,post}}{q_{pre,post}-1}}$$ (30)

$$RSE_{adapt}=\sqrt{\frac{SSE_{adapt}}{q_{adapt}-1}}$$ (31)
